# Case report: Lobular capillary hemangioma of the right main bronchus: a rare case with calcified and cystic lymph nodes

**DOI:** 10.3389/fmed.2025.1501312

**Published:** 2025-02-12

**Authors:** Xu-ping Chen, Jia Zeng, Wei Zhang, Peng Wu, Yan-ling Zhao, Zhi-shu Li, Ling Shen, Shi-xu He

**Affiliations:** ^1^Department of Respiratory and Critical Care Medicine, Guangyuan Central Hospital, Guangyuan, Sichuan, China; ^2^Department of Respiratory and Critical Care Medicine, Affiliated Hangzhou First People’s Hospital, School of Medicine, Westlake University, Zhejiang, China

**Keywords:** lobular capillary hemangioma, LCH, pyogenic granuloma, PG, argon plasma coagulation, APC, Infantile hemangiomas, IH

## Abstract

Lobular capillary hemangioma (LCH), typically a benign vasoproliferative lesion of the skin or mucosal surfaces, is exceptionally rare in the trachea. Now, we present the second reported case of LCH found in the right intermediate bronchus, characterized by calcification within the lesion and cystic changes. These distinctive features should alert clinicians to consider LCH in the differential diagnosis of other benign vascular tumors and mediastinal lymphadenopathy, particularly when calcified and cystic lesions are observed.

## To the editor

**FIGURE 1:**
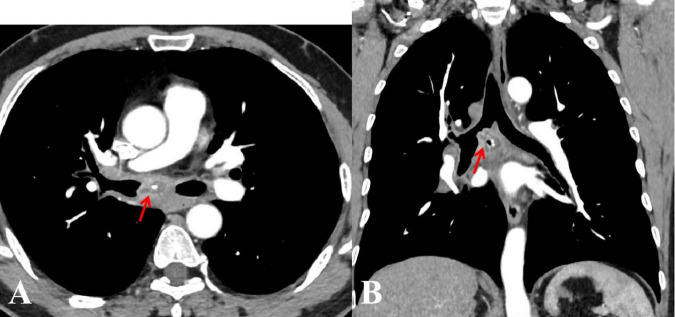
Enhanced chest computed tomography axial **(A)**, and coronal **(B)** views revealed a lobulated mass in the right main bronchus, enlarged mediastinal lymph nodes, and possible necrosis with “focal calcifications” (*arrows*).

**FIGURE 2:**
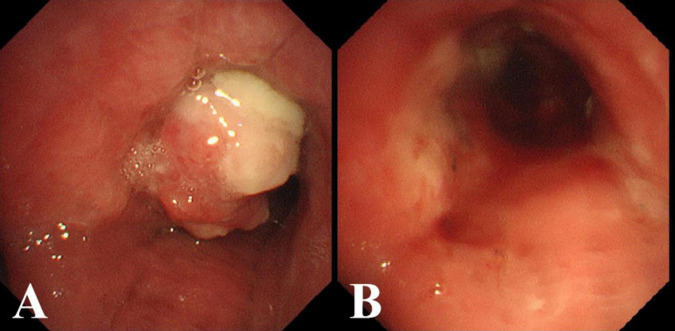
**(A)** Bronchoscopic view of the mass occluding the right main bronchus. **(B)** After excising the mass with an electrocautery snare and applying argon plasma coagulation (APC).

A 57-year-old male nonsmoker, previously in good health, presented with a cough, white sputum, and occasional minor hemoptysis following cold-like symptoms without fever and chills, 40 days prior. He had no history of regular medication use and worked in construction, often exposed to dust. Physical examination revealed normal lungs and heart. Routine lab tests were unremarkable. An enhanced chest CT scan revealed a lobulated mass in the right main bronchus with subcarinal and right hilar lymph node involvement, and possible necrosis and “focal calcifications” (Figures 1A,B). Bronchoscopy revealed a large, smooth-surfaced polypoid mass in the right main bronchus obstructing approximately 80% of the airway (Figures 2A,B). The mass was resected using an electrocautery snare and argon plasma coagulation (APC) was applied. A malignant tumor was highly suspected, clinically. Grossly, the mass was irregular in shape, measuring 2.0 × 1.0 × 0.9 cm, with a visible cystic area of 1.0 × 0.9 ×0.4 cm on the section. Microscopically, many neutrophils and some plasma cells were observed infiltrating the interstitium beneath the bronchial mucosa, and there was a significant proliferation of small blood vessels forming lobular structures. Immunohistochemical staining of tumor tissue indicates the presence of vascular markers such as CD31 (+), CD34 (+), Factor VIII (+), SMA (+), Desmin (−), S100 (−), CD99 (partly+), BCL-2(−), and Ckpan (AE1/AE3) (−). In this biopsy tissue, a large number of neutrophils were found in the subepithelial stroma of the bronchial mucosa

with no evidence of malignancy. Therefore, infection needs to be ruled out first, and targeted NGS (tNGS) of the tissue specimen for pathogens was further conducted.

**FIGURE 3:**
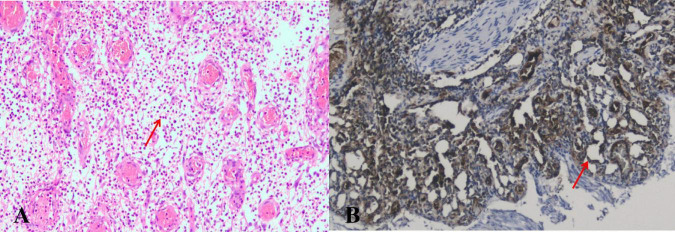
**(A)** The microscopic view of the mass reveals a significant number of neutrophils and a few plasma cells infiltrating the bronchial submucosal interstitium (*arrows*). **(B)** Under the microscope, a significant amount of lobular small blood vessel proliferation can be observed, with no cancer cells present (*arrows*) (CD31 positive staining, ×100).

The results of tNGS identified Streptococcus constellations in the tissue paraffin section with 2,144 sequences, accounting for 8.82% of the total. However, since the patient’s inflammatory markers are not elevated and the tissue specimen sent for examination is not completely sterile, we do not consider the airway mass to have been caused by an infection. LCH was finally diagnosed based on the patient’s pathological results (Figures 3A,B). A third-party remote pathological consultation also confirmed the pathological diagnosis of LCH. The patient has been recurrence-free for over six months.

## Discussion

LCH, also known as pyogenic granuloma (PG), is a benign vasoproliferative tumor usually found on the skin or mucosal surfaces (1). Primary tracheal tumors are rare, with approximately 2.7 new cases per year, and are typically malignant in adults (2) {#2}. A tracheal origin for LCH, however, is exceedingly rare, with only a few case reports in the literature (3, 4). Furthermore, LCH in the bronchus is even rarer, which are often misdiagnosed as asthma or bronchitis due to symptoms like cough, hemoptysis, and wheezing. So far, only a few cases have been reported in the bronchus intermedius, one of which was a Chinese case who also initially presented with cold-like symptoms and enlarged mediastinal lymph nodes, similar to the case we have presented (5). The etiology, presentation, behavior, and management of LCH are not yet fully understood. Limited research indicates that mutations in *GNAQ p. Arg183Gln*, *BRAF p. Val600Glu*, and *HRAS* have all been found to be associated with the mitogen-activated protein kinase (*MAPK*) signaling pathway, providing supporting evidence for key driving factors in the pathogenesis of PG (6, 7). Unfortunately, in this case, next-generation sequencing was not utilized to screen for potential mutations due to the patient’s financial constraints. Other theories suggest LCH may result from local irritation and minor trauma, neovascular response to an angiogenic stimulus with an imbalance of promoters and inhibitors, or viral and bacterial infections (8). Since LCH is commonly found in areas susceptible to trauma, such as the skin, it is quite common to see ulceration and coexisting chronic inflammation in the stroma (9). However, in all reported cases of tracheal LCH, there was no history of trauma noted. No trauma, such as tracheal intubation or foreign body aspiration, was identified in the case we reported. Therefore, it is still unknown whether trauma can cause tracheal LCH. Previous studies have reported numerous cases of drug-induced PG, such as retinoids, antiretroviral drugs, chemotherapy drugs, biologics, immunosuppressants, and others (10–13) {#8}. The patient in this case, however, denied having any underlying diseases or a history of medication use, which suggests a very low possibility of drug-induced PG. Although coughing and hemoptysis are commonly reported in most cases of tracheal PG, cold-like symptoms are rarely reported. The case discussed exhibited cold-like symptoms at the onset of the illness, and streptococcus constellatus was detected in the LCH lesion. Thus far, there have been no reported cases of PG induced by infection. It is unknown whether colds or bacterial infections could trigger tracheal LCH, like trauma. The imaging features of the discussed case are unique. As of now, only one case of LCH involving a lesion in the right main bronchus and mediastinal lymph nodes has been reported, while the remaining cases have all been in the trachea without any lymph node involvement (5). Herein, we are presenting another case that also involved the right main bronchus, along with the additional involvement of the mediastinal and right hilar lymph nodes, which were fused with the tracheal lesion. The differential diagnosis of this case necessitates consideration of several diseases. Differential diagnoses include vascular tumors like infantile hemangiomas (IH), which typically present in infancy and resolve, often GLUT1-positive (14, 15). This case does not align with the clinical manifestations of IH; therefore, the diagnosis is not being considered. This case also needs to be distinguished from myopericytoma in terms of pathological manifestations. A key characteristic of myopericytoma under the microscope is that tumor cells are arranged in a concentric pattern around blood vessels, and the tumor cells often express markers positive for myogenic origin (16). However, upon closer examination, the presentation of this case under the microscope is not consistent with myopericytoma. Conditions causing mediastinal lymphadenopathy, such as lymphoma, sarcoidosis, and metastatic carcinoma, were also considered (17). After all, LCH is an extremely rare cause of mediastinal lymphadenopathy. “Popcorn” calcification in LCH, first described by Kalanjeri et al., which was considered unique and aids in differentiating calcified endobronchial lesions (18). We are now reporting a second case of calcification in LCH distinct in shape and location. Meanwhile, cystic lesions that were mistakenly identified as necrosis on enhanced CT imaging have not been previously described in cases of LCH. These findings in this case were unique and should prompt clinicians to consider LCH in the differential diagnosis of mediastinal lymphadenopathy, particularly with calcified and cystic lesions. Treatment for tracheobronchial LCH primarily involves biopsy forceps, cryotherapy, and electrocautery snare, similar to skin or mucous LCH (19). Prakash et al. also reported a case of a pregnant woman with a large tracheal LCH lesion who experienced life-threatening respiratory failure resembling severe asthma and was successfully treated with surgery (2). Bronchial artery embolization (BAE) and extracorporeal membrane oxygenation (ECMO) have been reported to assist in the surgery of tracheal LCH (2, 20). Regardless, the overall prognosis for various treatment methods is generally positive (5). An oral LCH recurrence rate of 16% has been reported, which is believed to be the result of failure to remove etiological factors, incomplete excision, or re-injury to the area (21). As of now, most reported cases of airway LCH recurrence are rare, except for the case of the pregnant woman described earlier. Reports of recurring cases of LCH are extremely rare; however, most cases have a follow-up duration of less than 2 years (22). Furthermore, optimizing the treatment of tracheobronchial LCH still requires long-term observation and more controlled clinical trials. Currently, research on tracheobronchial LCH is primarily focused on case reports, while research on the pathogenesis of the disease is extremely limited. Furthermore, more long-term clinical comparative studies on airway LCH are necessary.

## Data Availability

The original contributions presented in the study are included in the article/supplementary material, further inquiries can be directed to the corresponding author.
